# Pericapsular Nerve Group Block Versus Lumbar Epidural Block for Pain Management After Hip Surgeries with a Focus on Pediatric Patients: A Narrative Review

**DOI:** 10.3390/neurolint17090142

**Published:** 2025-09-08

**Authors:** Shahab Ahmadzadeh, Hunter M. Schwab, Mary O’Dell Duplechin, Kalob M. Broocks, Jon D. Hirsch, Joseph Drinkard, Sahar Shekoohi

**Affiliations:** 1 Department of Anesthesiology, Louisiana State University Health Sciences Center at Shreveport, Shreveport, LA 71103, USA; 2 School of Medicine, Louisiana State University Health Sciences Center at Shreveport, Shreveport, LA 71103, USAmmo002@lsuhs.edu (M.O.D.);

**Keywords:** pericapsular nerve group block, lumbar epidural block, hip surgeries, pain management

## Abstract

Pediatric hip surgeries are associated with moderate to high levels of pain, which, in severe cases can lead to opioid prescription and use. There is a growing focus on reducing post-operative pain in these patients to decrease the need for opioids, as well as increase early mobilization for recovery. Conventional methods of pain relief using opioids can have unwanted negative impacts on pediatric patients such as respiratory depression, nausea, confusion, and the concerning possibility for the development of dependence. Likewise, traditional methods of anesthesia, like the lumbar epidural block, can have unwanted systemic side effects, such as hypotension, urinary retention, arrhythmias, and spinal abscesses. These complications can lead to longer hospital stays and delayed recovery. This review analyzes the efficacy of a newer regional anesthesia technique, the pericapsular nerve group (PENG) block, in comparison to the lumbar epidural block. This technique utilizes precision-based anesthesia to selectively block the articular branches to the hip joint while avoiding the main trunks of the femoral and obturator nerves. Additionally, with the utilization of high-resolution ultrasound to guide the blocks, providers can increasingly count on proper insertion and predictable anesthetic spread. The result is a motor-sparing blockade that shows promise in allowing earlier mobilization and better functional recovery times after pediatric hip surgeries.

## 1. Introduction

### 1.1. Overview of Pediatric Hip Surgeries

Historically, pediatric hip surgery is an area of medicine that has seen minimal research in relation to attempted advancements in surgical anesthesia techniques for hip surgeries. This is likely in part due to the lower prevalence of hip surgeries in pediatric populations, who have far fewer risk factors for the development of hip disease. This is in comparison to older populations, especially females over 65, who have numerous factors for the generation of degenerative processes and fractures of the hip (osteoarthritis, osteoporosis, age-related wear and tear) [1,2]; however, hip surgery can be required to address issues stemming from developmental conditions in certain pediatric patients. This would include cases such as developmental dysplasia of the hip, Legg–Calvé–Perthes disease, and slipped capital femoral epiphysis, all of which can lead to both short and long-term complications if not diagnosed and managed properly [3]. Additionally, traumatic etiologies with resultant dislocations and fractures can certainly necessitate urgent hip surgery in pediatric populations. Although rare, proper realignment and management of any vascular disruptions is critical in preventing negative outcomes, such as avascular necrosis.

### 1.2. Postoperative Pain Issues

Pediatric hip surgery can have high levels of associated post-operative pain, which can become problematic for recovery times after surgery. Effective post-operative pain control in patients undergoing hip surgery is essential to the outcomes for these patients. Around one in five pediatric patients has been found to experience short-term pain, defined as significant pain still being present at two weeks post-surgery [4]. Improper pain control has been associated with increased length of hospital stays and longer overall recovery times to regain adequate mobility. The monetary burden associated with these longer hospital stays can lead to financial strain for the parents of these children, with socioeconomic factors such as poor credit and debt being directly attributed to prolonged hospitalization [5,6]. As a result, significant post-operative pain can have multifactorial effects that ultimately result in both physical and psychological consequences for the children involved. Although similar effects can be seen in any age group, these appear to be especially important in the pediatric population. For example, it is of note that a study by Daughtrey et al. found that increased duration of hospital stay was positively correlated with incidence of mental health disorder diagnoses one year after surgery [7].

### 1.3. Introduction to Lumbar Epidural Block vs. Pericapsular Nerve Group Block

Opioid-sparing pain management techniques reduce concerns over common adverse effects seen with opioids, such as constipation, nausea, and drowsiness. This is especially significant in pediatric populations that have less predictable responses to these high-potency medications. It also eliminates the chance for improper prescribing and abuse, which has become an area of increasing concern. Although the prescribing of these controlled substances is more closely monitored now than it was historically, one study found that 46% of pediatric opioid prescriptions were categorized as high risk [8]. As a result, there is growing interest in comparing newer regional anesthesia techniques with the more traditional approaches. One such case is the comparison of the lumbar epidural block to the pericapsular nerve group (PENG) block. The lumbar epidural block has historically been a popular technique of regional anesthesia for pediatric hip surgeries. The introduction of ultrasound guidance and subsequent refinement of the technique has greatly improved the reliability of epidural injections, providing reproducible results over the past 20 years [9].

In recent years, the PENG block has gained popularity since its introduction by Girón-Arango et al. in a paper describing the use of this novel technique in relation to 5 patients who underwent surgery for hip fractures in 2018 [10]. The PENG block is proposed to offer a more refined method of analgesia via the targeting of specific nerves of the anterior capsule of the hip [11]. The motor sparing ability of this block, in addition to various other beneficial aspects of the block, shows promise as a potential innovation in the standard of care for post-operative pain relief for pediatric patients. In this review, we will discuss the comparative efficacy of PENG blocks compared to lumbar epidural blocks for pain management after hip surgeries.

## 2. Methods

The literature search involved a comprehensive search of the databases PubMed, Google Scholar, and JSTOR using the exact Boolean terms: (“hip surgery” or “hip replacement” or “hip fracture” or “hip arthroplasty” or “hip arthroscopy”) and (“PENG” or “Pericapsular nerve group block”) and (“lumbar epidural block” or “lumbar block”). Eligible studies included randomized controlled trials, cohort studies, case–control studies, and case reports, whereas conference abstracts, letters, and animal-only studies were excluded. The literature search was confined to material published from March 2003 until June 2025. The population of interest was pediatric patients, defined as adolescents and children less than or equal to 18 years of age, undergoing hip-related surgery. The surgeries included were (1) total or partial hip arthroplasty, (2) hip arthroscopy, (3) developmental dysplasia of the hip surgeries (both open and closed reductions), and (4) hip fracture repairs. Both unilateral and bilateral surgeries were considered for each of these procedures. The intervention examined was the PENG block, with only the single-shot technique considered. The comparator was the lumbar epidural block, in which both single-shot and continuous infusion techniques were considered. Other regional block methods, such as the fascia iliaca and femoral blocks, were not included in this review. The primary outcomes of interest in this review were post-operative analgesia, as measured by pain scores, time to ambulation, and opioid use. Additionally, secondary outcomes of adverse effects and complications, such as motor blockade and urinary retention, were evaluated.

## 3. Pericapsular Nerve Group (PENG) Block

As stated, the PENG block offers targeted analgesia for hip pathology, specifically of structures found within the anterior capsule of the hip compartment. This makes it an appealing alternative to lumbar epidural blocks, especially in pediatric populations. The PENG block allows for motor-sparing analgesia, ideal for children requiring early postoperative mobilization. Studies determined that the PENG block is a reliable method for managing pain in the early postoperative period, promotes quicker motor function restoration, and reduces the need for opioids [12].

The PENG block is unique in that it selectively anesthetizes the articular branches to the hip joint while avoiding the main trunks of the femoral and obturator nerves. The articular branch of the femoral nerve supplies the anterior portion of the hip joint, and the articular branch of the obturator nerve innervates the anteromedial hip capsule. In addition to the articular branches of the femoral nerve, the anterior hip capsule is innervated by the obturator nerve and accessory obturator nerve. The accessory obturator nerve, found in roughly 10% to 30% of individuals, typically arises from the L3 and L4 nerve roots and commonly provides innervation to the hip joint and the adductor longus muscle [13]. The articular branches that supply the anterior hip capsule converge near the iliopubic eminence, where they travel near the iliopsoas muscle and tendon. Studies have shown that the PENG block may offer comprehensive hip pain relief by targeting the articular branches, potentially avoiding motor nerve blockade [14]. Due to these branches converging near the iliopubic eminence, the PENG block involves injecting the local anesthetic into the fascial plane located between the psoas muscle and the superior pubic ramus [15]. This is commonly performed under the guidance of ultrasound to ensure proper orientation and minimize the chance of improper placement of injection. Children have less muscle mass and smaller bony landmarks. This can both aid and complicate ultrasound identification. The proximity of neural and vascular structures in smaller patients increases the importance of precise needle placement to avoid systemic toxicity. The PENG block is typically administered with the patient in the supine position. Depending on the child’s size, either a high-frequency linear probe (5–10 MHz) or a curvilinear probe (3–8 MHz) may be required to visualize the nerve effectively [16]. In smaller pediatric patients or thin individuals, a high-frequency linear probe (6–13 MHz) may be used because it provides better resolution of superficial structures, which is sometimes necessary due to their smaller anatomy [12]. Therefore, curvilinear is ideal for depth, while linear is better for clarity in superficial anatomy. The femoral artery and the femoral head in deeper views are both used to help properly orient during the block. Once these two landmarks have been identified, the probe can be manipulated to bring both the anterior inferior iliac spine and the iliopubic eminence additionally into view. Once this visual has been obtained, injection of local anesthetic can be performed properly along the subfascial plane [17]. Visualizing the elevation of the psoas tendon after injection also helps confirm that the proper fascial plane was achieved. The spread is mostly limited to the previously stated area between the psoas muscle and the superior pubic ramus, which is advantageous in avoiding unnecessary motor blockade. It has been found that 0.3–0.5 mL/kg of 0.2% ropivacaine, but not exceeding 20 mL total, is the supposed optimal agent and dosing for pediatric patients [18]. Any amount of anesthetic exceeding this threshold has been associated with increased levels of anesthetic spread, which can lead to unwanted motor blockade, negating the major benefit of this block compared to other methods of anesthesia [12]. When performed correctly, this motor-sparing aspect is helpful for pediatric procedures, where early mobilization is associated with improved outcomes. With appropriate ultrasound guidance and probe selection tailored to patient size, the PENG block can be safely and effectively integrated into pediatric anesthesia protocols.

**Figure 1:**
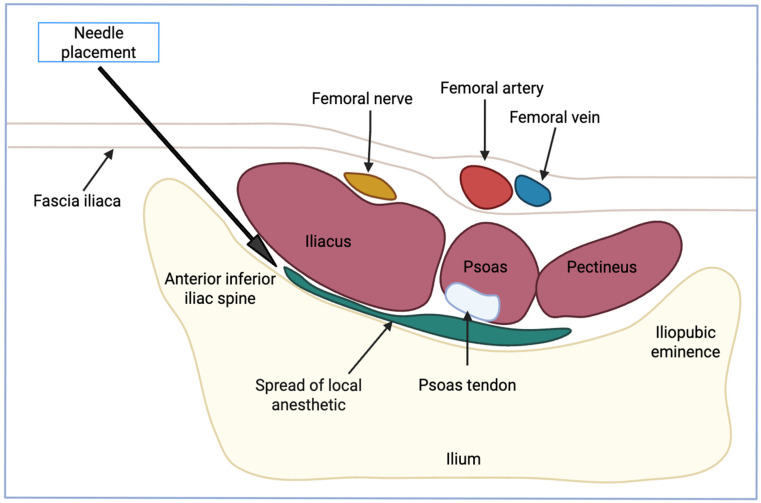
Anatomy of PENG block.

A scarcity of published literature is available regarding the average duration of analgesia in the pediatric population specifically, but some recent studies have demonstrated the analgesic effects of this block in the general population. The average duration of analgesia in this block, as measured by time to first rescue analgesia request, was found to be about 6.6 h. Although some case reports indicate potentially lengthened durations of analgesia, the stated timeframe of 6.6 h is in concordance with a randomized controlled trial from 2024. In this study, pediatric patients undergoing elective unilateral hip replacement received 0.25% bupivacaine at a dosing of 0.5 mL/kg [19]. Additionally, the complementation of the PENG block with adjuvants has been shown to improve early post-operative pain ratings when compared to the PENG block alone. The addition of a Lateral Femoral Cutaneous Block (LFCB) has been shown to significantly increase the time to first opioid request from 10.9 h in patients receiving solely PENG block, compared to 15.3 h in patients that also received LFCB during surgical treatment of hip fracture. Additionally, total tramadol administration in the first 24 h was also found to be significantly decreased [20]. Similar to adjuvant treatment with additional blocks, the supplemental administration of 8 mg of dexamethasone was shown to increase time to first rescue analgesic (445 min) when compared to the administration of 20 mL of 0.5% ropivacaine alone (389 min) in patients undergoing hip fracture treatment [21]. See Figure 1 below.

## 4. Lumbar Epidural Block

Lumbar epidurals are commonly used for lower body surgeries or to manage postoperative pain, especially in procedures involving the hips, pelvis, or lower limbs. Unlike general anesthesia, which affects the whole body, a lumbar epidural works locally and selectively by blocking nerve signals in targeted spinal regions. The lumbar epidural block targets the spinal nerve roots emerging from the lumbar and sacral regions. Administering a lumbar epidural block in pediatric patients requires an in-depth understanding of the developing spine. In neonates and infants, the conus medullaris extends to approximately the L3 vertebral level, with some variance noted, as compared to the L1 level in most adults. This slight variation brings with it a minimal increase in degree of difficulty ascertaining the termination of the conus medullaris on a patient-by-patient basis in pediatrics. With this comes the slightly increased likelihood of adverse effects associated with improper injection level. In children, the cervical vertebrae are underdeveloped compared to adults. This relative underdevelopment manifests as vertebrae consisting mostly of cartilage in younger pediatric patients before the cartilage is gradually replaced by mature bone. Additionally, the joints between the vertebrae are less pronounced, and the neck ligaments are generally weaker than those in adults [22]. This offers less resistance to needle advancement. Therefore, it is important to select appropriate intervertebral spaces for needle insertion to avoid spinal cord injury. The incorrect placement of a needle could lead to severe neurological deficits, such as motor paresis and hypoesthesia.

Caudal epidural blocks are widely used in pediatric anesthesia and are performed via a single injection through the sacral hiatus, offering an alternative to lumbar epidural techniques that often require catheter placement [23]. This approach is technically less invasive and may reduce risks associated with catheter-based lumbar epidurals. Ultrasound guidance, increasingly used in pediatric practice, enhances the accuracy and safety of caudal block placement, improving first-puncture success and reducing complications such as vascular puncture or subcutaneous injection [24]. Duration of analgesia after single-shot caudal block tends to be longer in younger children than in adults due to differences in anatomy and myelination, though it remains shorter than continuous epidural techniques. One study in the pediatric population showed caudal analgesia lasting 3.45 h for single caudal and 7.85 h for double caudal [25]. Adjuncts like clonidine or dexmedetomidine can extend this effect [26]. This range is comparable to that achieved with single-shot PENG blocks. However, the cephalad spread of anesthetic via the caudal route can be unpredictable, potentially leading to bilateral motor blockade, which may be undesirable in certain clinical scenarios [23].

**Figure 2:**
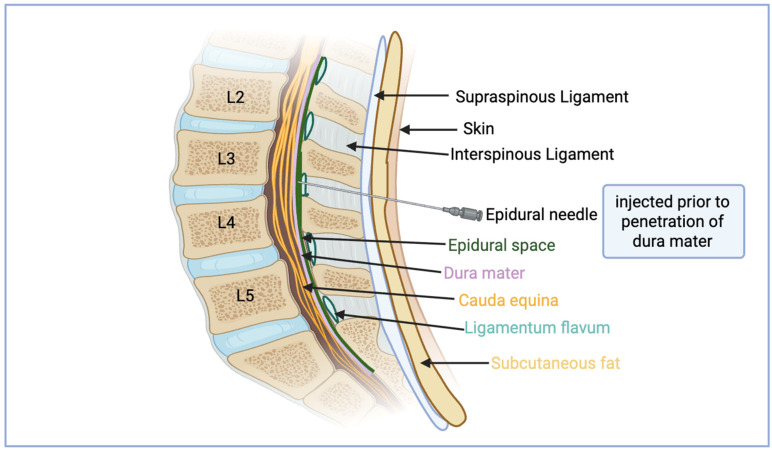
Anatomy of lumbar epidural block.

Bupivacaine and ropivacaine are commonly used local anesthetics in pediatric epidural anesthesia. Local anesthetics block voltage-gated sodium channels, preventing nerve impulse conduction and muscle contraction. Out of these two widely used agents, bupivacaine is considered to carry a higher risk profile. This higher risk profile stems from the increased incidence of ventricular arrhythmia complications due to its slow unbinding [27]. Local anesthetics target and block the open state of voltage-gated sodium channels, reducing or stopping signal transmission in vascular smooth muscle, which causes the muscle to relax [27]. The block can be performed with the patient either sitting or lateral decubitus, ensuring spinal flexion. Adequate spinal flexion is a necessary step to guarantee adequate intervertebral widening for proper needle insertion during the block. In the lateral position, the patient should curl up by pulling their knees toward their chest [28]. On insertion, the needle passes through several layers, including the skin, subcutaneous tissue, supraspinous ligament and interspinous ligaments, and finally the ligamentum flavum. As opposed to spinal blocks, the dura mater of the spinal cord is not penetrated. A commonly used technique to ensure proper placement of the needle and to avoid penetrating vital structures, practitioners are trained to gently feel for a loss of resistance to insertion. This loss of resistance signifies the needle has fully traversed the interspinous ligament and has entered the epidural space. Anatomically, the dura mater forms the internal border of the epidural space, with the interiorly located arachnoid mater and pia mater associated with the spinal cord. Within the spinal column, borders of the epidural space are defined by the posterior longitudinal ligament anteriorly, the ligamentum flavum and the vertebral lamina posteriorly, and the intervertebral foramina with its associated spinal nerve roots laterally [28]. In most pediatric patients the distance from the skin to the epidural space is about 22 mm at the lumbar region, as measured by MRI [29]. This would also apply to many children due to their small size. Once the space is identified, the provider may inject 5 to 10 mL of saline to gently open the epidural space and reduce the risk of puncturing blood vessels [30]. Ultrasound guidance provides clear imaging of nerves and surrounding anatomy needed for performing neuraxial and peripheral nerve blocks, which is especially helpful in children due to their shallow anatomical structures. The most frequent issue in pediatric regional anesthesia is an unsuccessful block [31]. The duration and efficacy of lumbar epidural analgesia are influenced by the choice of local anesthetic, its concentration, and the use of adjunctive medications. In pediatric patients, 0.5–1.0 mL/kg of 0.25% bupivacaine or 0.3% ropivacaine is generally given adjunctively with a form of general anesthesia [31]. Given the nature of the epidural block, anesthetic is able to spread locally both cranially and caudally along the axis of the spinal cord’s epidural space, with greater spread generally being noted in the caudal direction as a consequence of gravitational effects [31]. The major nerves targeted with an epidural block for hip surgeries include the femoral, obturator, and lateral femoral cutaneous nerves; however, the nonspecific targeting of both the dorsal and ventral rami as they leave the spinal canal causes loss of both motor and sensory modalities of the nerve root levels associated with these nerves. The commonly published nerve root levels for these nerves are L2–L4 for the femoral nerve, L2–L4 for the obturator nerve, although L2, L3 has been stated, and L2, L3 for the lateral femoral cutaneous nerve [32,33,34]. A randomized controlled trial by Casati et al. [35] looked at time to onset of analgesia, as well as duration of analgesia in patients receiving epidural anesthesia with 0.5% levobupivacaine, 0.5% bupivacaine, or 0.5% ropivacaine for elective hip surgery. Findings included onset of sensory block at 31 min, 25 min, and 30 min for each of the respective analgesics, with median dosages of 15 mL across all three blocks. Additionally, recovery of pinprick sensation was then found to occur at 214 min with levobupivacaine, 213 min with bupivacaine, and 233 with ropivacaine, which indicated similar duration of pain relief across the major analgesic agents used in lumbar epidural administration. Overall, lumbar epidural blocks provide effective analgesia for various surgical procedures in pediatric patients. When administered with careful consideration of the unique anatomical and physiological characteristics of children, this technique offers significant benefits in pain control and patient comfort. See Figure 2 below.

## 5. Comparison of the Efficacy Between Blocks

As previously stated, the desirable effects of the PENG block are due to its supposed specificity towards the sensory fibers of the anterior hip capsule to inhibit or decrease opioid use after surgery. In particular, the PENG block provides its analgesic effect on the obturator, accessory obturator, and sensory capsular branches of the femoral nerve [18]. Subsequently, its effects are limited to sensory information of the hip and proximal thigh. Typically, the procedure is unilateral, with the hip being operated on receiving injection, but bilateral PENG blocks can be used in more complex cases if both hips require surgical intervention. Common indications for this block include hip fractures or developmental and congenital dysplasia of the hip [12]. However, this procedure has also been used in a variety of clinical settings for analgesia, such as vaso-occlusive crises in sickle cell disease, radiofrequency ablation for osteoarthritis analgesia, stripping and ligation of the saphenous vein, medial thigh surgery, and more, thus proving its efficacy outside of the hip-related area [36]. Decreasing the use of opioids after surgery is a serious concern for post-operative patient care. Opioid use can lead to nausea, delirium, respiratory depression, vomiting, constipation, and addiction. The PENG block has been shown to decrease opioid use after surgery when compared to other analgesic techniques, like the Fascia iliaca compartment (FI) block and sham trials [18,37]. This is a major finding, as opioid-sparing care is the gold standard when dealing with pediatric patient populations. In addition to decreasing opioid use, the block has also shown an increased time to first opioid use after surgery, reduced pain scores during motion and rest, and improved functional recovery [29]. Another positive aspect of the PENG block is decreased systemic side effects. Compared to the lumbar epidural block, which can include serious side effects like hypotension, meningitis, abscesses, hematoma formation, renal puncture, and high neuraxial anesthesia, the PENG block has not been indicated to increase the risk of these adverse effects [18]. The limited available data on this block in pediatrics has shown no documentation of serious adverse effects, such as mobility disorders, or systemic toxicity (seizure/arrhythmia) [11,19]. There is the theoretical concern for quadriceps motor blockade with inadvertent injection spread or high injection pressure, but there have been no documented cases in pediatric patients [12]. The only findings of note are from a randomized-controlled trial that included 28 patients receiving PENG block, of which there were two instances of post-operative nausea, and one instance of localized hematoma [19]. Future prospective studies are needed to further substantiate the validity of this information.

The disadvantages that surround the PENG block begin with the lack of research completed on the pediatric population as compared to the general population. The PENG block in pediatrics is a developing procedure, and as such, few large-scale studies have been performed to determine its efficacy. With this being said, the few clinical trials that have been carried out using this block in the pediatric population seem to show positive clinical outcomes. Another downside to the PENG block is that, while it is asserted to only affect sensory fibers, there have been documented instances of quadriceps weakness after use [12,18,38]. Motor blockade is typically seen in PENG blocks that use an increased amount of local anesthetic, above the 20 mL limit that has been posed as the maximum tolerable dosage in some literature. The inhibitory motor effects are believed to stem from the unintentional anteromedial spread anesthetic away from the local injection site and down the Psoas Major muscle [38]. Factors that contribute to the medial movement of local anesthetic include a high injecting pressure, high injecting volume (above 20 mL), and rupture of the iliopectineal bursa. There is also increased local anesthetic systemic toxicity, neurotoxicity, and cardiotoxicity seen in infants and young children when compared to adults; increased caution is advised when treating this patient population [12]. Finally, most PENG blockades are given as single-shot injections with anesthetic given in a one-time dose. This can lead to limited analgesic time and rebound hyperalgesia when compared to the continuous infusion modality afforded in various other blocks. To avoid this outcome, systemic steroids can be used to prolong analgesia with supplemental NSAIDs (acetaminophen and ketorolac) to prevent rebound pain [15].

In comparison, the lumbar epidural block results in a bilateral analgesic effect by non-specifically targeting the motor and sensory nerve roots that leave the spinal cord at the intervertebral foramina. This type of block generally uses an epidural catheter to allow for continuous analgesia, thus increasing the overall duration of post-operative analgesia [39]. Its continuous infusion of local anesthetics is advantageous in that it allows for a broader and extended coverage of analgesia needed for more severe cases. The efficacy of lumbar epidural block in respect to pain management is best seen in lower abdominal surgeries over thoracic and upper abdominal surgeries, respectively [39]. Just like the PENG block, the lumbar epidural block also results in decreased opioid use, in relation to initial dosage and overall frequency, leading to better post-operative outcomes. For example, patients who received epidural blocks over lumbar plexus blocks were found to cut opioid use in half, 2 mg/kg compared to 4 mg/kg, over the first 48 h postoperatively [40].

While the continuous infusion seen in lumbar epidural blocks can have positive effects, there is a small subset of data on epidural anesthesia in pediatric cases. Continuous analgesia is challenging in children due to imprecise pain assessment seen at different ages, technical difficulty upon entering and removing the catheter, drug dosing, and lack of expertise [39]. Also, with this block, there is an increased risk of major adverse effects compared to PENG. This includes systemic complications such as hypotension, urinary retention, arrhythmias, and spinal abscesses. These adverse events can lead to prolonged hospital stays for affected patients until their management is stabilized; however, although incidence rates of these serious complications vary depending on the study, the risk is generally considered to be minimal when taking the vast majority of cases into account [41]. Catheter kinking, pruritis, premature catheter removal, leaks, motor blocks, and nausea are all minor complications that are seen more in daily clinical practice [39]. Finally, increased caution should be used when treating infantile patients because there is increased risk of complications in children under six months of age [41].

It is also worth noting that PENG and lumbar epidural blocks are both preferred to be placed under general anesthesia in pediatric patients. In the case of lumbar epidural blocks, pediatric patients are often unable to reliably communicate symptoms that could indicate improper needle placement. Additionally, they commonly struggle to tolerate the awkward lateral decubitus positioning and process of needle advancement without strain. This is substantial, as even small degrees of movement could cause severe complications, due to the proximity of the intended needle placement to important neural structures. As a result, it is widely accepted that placement of epidurals under general anesthesia with close monitoring is the best clinical practice in pediatric populations [42]. There are a few exceptions where lumbar epidurals can be considered based on clinical judgment and comorbidities. For example, patients who have severe respiratory disease or are hemodynamically unstable could opt for awake placement of the block, as long as they are old enough to cooperate. This can avoid risks associated with the administration of general anesthesia, while still providing analgesia [43]. This is in contrast to the recommended guidelines when placing the same block in an adult, where the feedback offered, such as pain or elevated pressure, can aid the physician in avoiding excessive puncture and unintended damage. For similar reasons, PENG block is also preferred to be placed under general anesthesia in pediatric patients. Although the risk of serious adverse effects is less significant, there is still an increased risk of inadvertent movement, due to discomfort from awkward posturing for block placement, in the case of an awake patient. This can lead to difficulty localizing necessary structures on ultrasound, and ultimately improper injection of anesthetic. Consequently, a systematic review in the Journal of Clinical Medicine indicated that PENG block, being performed under general anesthesia, should remain the overall standard of care [43]; however, there is evidence that blocks analogous to the PENG block have been beneficial when performed on awake patients under certain circumstances. For example, a trial on ultrasound-guided fascia iliaca compartment blocks found the block to provide superior analgesia to IV morphine in children suffering from fractures of the femur [44]. This indicates similar regional blocks, like the PENG, could be valuable in dealing with severe pain control pre-operatively in a hospital setting, although further studies are needed.

Total hip arthroplasty accounts for USD 15 billion annually due to cost of procedure, lengthened hospital stay, and post-operative opioid consumption. Reducing factors that attribute to these outcomes can help people reduce cost for this procedure. Factors that can affect overall patient experience and recovery are symptoms like nausea, post-operative opioid use, time to first ambulation, and length of hospital stay. All these factors, as well as others, can not only change a patient’s physical and mental recovery after a major surgery but also their cost of hospitalization. A study from Remily et al. showed an average length of hospital stay of 39.7 h respectively after a total hip arthroplasty with PENG block. Remily et al. also had 85% of their patients be discharged home and only 15% discharged to a rehabilitation facility. Time to first ambulation in the PENG group was averaged to about 13 h [45]. Postoperative nausea has rarely been documented outside of case reports for PENG block in pediatrics. Current literature also indicates that the use of dexamethasone as an adjunct shows promise in minimizing this complication further [11]. Additionally, a retrospective audit of over 3000 lumbar epidurals performed over 15 years found a complication rate of about 0.76%. Neonates and infants were found to be at greater risk, with complication rates of 4.2% and 1.4% respectively, compared to older children, who saw a complication rate of around 0.6%. The most common complications seen included dural puncture, local skin infections, and inadequate block [46]. Although rare, such complications were found to be essentially nonexistent with the PENG block [11,12,18]. While there is not direct evidence of cost and cost reduction between the two blocks, there are multiple ways that improved anesthesia can reduce the financial burden on patients. A couple ways the PENG block can help reduce out-of-pocket cost is by shortening hospital stay, opioid use, adverse events related to hospital stay, and rehabilitation status. See Table 1 below.

## 6. Discussion and Conclusions

Across our research we found evidence that the PENG block has promising effects on functional efficacy and decreased post-operative opioid consumption in pediatric patients. Current literature is consistently showing decreased opioid use and increased functional improvement after hip surgery in both adult and pediatric patient populations [12,18,37]. While PENG block may not provide the extent of analgesia that the lumbar epidural block can, there is reason to argue its effectiveness over the latter in certain situations. While there is no significant difference between the length of hospital stay after procedures [45], the evidence does seem to point towards a difference in the time to first postoperative opioid, with the PENG block having a longer interval [49]. This blockade also has a lower chance of significant complications that can follow the lumbar epidural block, such as urinary retention, hypotension, and nerve injuries [41]. Due to the intricacy of the lumbar epidural block in pediatric patients, the PENG block may turn out to be the overall safer and more viable option in many cases.

With this being said, there are a number of factors that play a significant role when deciding the most effective method of analgesia for pediatric hip surgery patients. The many complexities that contribute to this decision in pediatric hip surgeries include aspects such as invasiveness of the procedure, desired duration of analgesia, anatomical variation by developmental age of the patient, and risk of associated complications. When considering how these aspects can drastically change from patient to patient, it is hard to truly point out one method of anesthesia as being broadly superior to the other; however, it is more accurate to describe these two blocks in relation to where they fall on this overall spectrum. The investigated literature pointed to the lumbar epidural block as a generally better mode of anesthesia when dealing with severe pathologies [39]. This is due to these more invasive surgical interventions requiring longer durations of analgesic relief for the patient. These factors point towards the lumbar epidural block as continuing to remain a standard of care in a subset of specific situations. Additionally, the caudal epidural block offers a less risky alternative to the traditional lumbar epidural block. It affords the ability to perform single injections of anesthetic under ultrasound guidance, as well as double injections if an increased analgesic effect is desired, while still maintaining desirable analgesic outcomes for patients; however, similarly to the lumbar epidural block, the cephalad spread of anesthetic in this technique can be unpredictable, with the undesirable potential for bilateral motor blockade. As a result, the PENG block was found to be sufficient for surgeries with lower levels of invasiveness and decreased expected post-operative pain.

In scenarios where the PENG block is indicated to provide a sufficient level of analgesia, the findings pointed towards several positive effects that improve post-operative patient safety and recovery. The stated anatomical specificity of the block and its resultant effects indicate that the PENG block could be a milestone in the management of pediatric hip surgery patients and post-operative pain analgesia. This is one of many examples in modern medicine where there is a shift being made towards more precision-based care. In this case, the motor-sparing ability and decreased risk of systemic side effects are what can lead to decreased time to active recovery in patients. Additionally, the associated shortened length of hospital stays is a secondary outcome of specific significance. These two factors could improve the ability of patients to regain full mobility while simultaneously leaving the families of these pediatric patients in better financial status.

Further research is needed to determine the accuracy of numerous findings in relation to both blocks. The literature on these blocks is scarce in pediatric populations, and it is difficult to make any significant takeaways from studies focusing on adult subjects, due to the substantial differences in anatomy and desired post-operative goals. This research could help determine any improvements that can be made in the method of analgesic delivery for the PENG block. Additionally, more research is needed to further determine the accuracy of the stated 0.3–0.5 mL/kg of 0.2% ropivacaine dosage that is currently accepted as the clinical standard. Due to the relative novelty of this approach, it is likely that more data will come to light as the block is continued to be used by more clinicians. In time, this will allow for definitive analysis of the efficacy of these blocks in relation to one another and the establishment of evidence-based guidelines for how they should be carried out.

Based on the available literature and the comparative points discussed above, Table 2 summarizes key clinical factors that may guide the choice between PENG, lumbar epidural, and caudal epidural blocks in pediatric hip surgery. While the focus of this review is on PENG and lumbar epidural techniques, caudal block is included for context as it remains a widely used option in children. These considerations integrate pain severity, surgical scope, motor-sparing priorities, and safety in patients with contraindications to neuraxial anesthesia.

Ultimately, block selection should be individualized, balancing the expected surgical pain, desired functional recovery, and patient-specific risk factors to achieve optimal postoperative outcomes in pediatric hip surgery.

## 7. Limitation of Current Evidence

One limitation worth mentioning is that there are very few high-quality studies directly comparing PENG, lumbar epidural, and caudal blocks in pediatric patients undergoing the same types of surgery. Much of the available evidence comes from case reports or is adapted from adult studies, which makes it difficult to draw firm conclusions for children.

In addition to this, while regional anesthesia techniques such as the PENG block and lumbar epidural block have been increasingly studied in adult populations, there remains a notable lack of high-quality data specific to pediatric patients. The current body of evidence for PENG block efficacy in pediatric hip surgeries is primarily limited to case reports, small case series, and extrapolation from adult studies. As a result, the available data do not meet the threshold required for a formal systematic review or meta-analysis, particularly regarding efficacy outcomes such as pain scores, opioid consumption, and recovery metrics in children.

Given these constraints, this review adopts a narrative format to synthesize existing knowledge, highlight emerging trends, and underscore the need for further high-powered, pediatric-specific research. We aim to provide a comprehensive educational resource that outlines current techniques, anatomical considerations, and early findings while acknowledging that robust comparative studies are needed to establish evidence-based guidelines for clinical practice in this population.

By summarizing the current literature and identifying key areas for future investigation, this review serves as a foundational reference for clinicians and researchers seeking to optimize post-operative pain management strategies in pediatric hip surgery.

## Figures and Tables

**Table 1:** Comparative Studies.

Author (Year)	Groups Studied and Intervention	Results and Findings	Conclusions
Domagalska et al. (2023) [18]	Case report of two children under the age of 5 to determine the analgesic effect of PENG block on hip surgery.	Neither patient required extra opioids during the surgery, or breakthrough opioids or muscle relaxants overnight. Both had numeric pain scores either at or below 3/10. Patients could participate in physical therapy the next day, and there was no proof of block complications.	PENG block provided opioid sparing management and retained motor functioning in these pediatric patients. Observations are encouraging for the efficacy and safety of PENG, but further studies are required.
Orozco et al. (2019) [47]	Case report of eight-year-old patient undergoing PENG block for open hip surgery.	Patient did not require intraoperative use of opioids in infusions or boluses. The patient had no pain up to 72 h postoperative recovery, had a pain level of 2/10, and did not require additional analgesia.	PENG block provided opioid sparing management along with decreased pain postoperatively. Observations are encouraging for the efficacy and safety of PENG, but further studies are required.
Domagalska et al. (2023) [48]	A Prospective, Randomized, Double-Blinded Clinical Trial with 468 patients to determine the effect of PENG block on pain management and recovery after total hip arthroplasty.	Compared to sham groups, PENG block had decreased overall opioid consumption, increased quadriceps strength, increased time to first opioid, and decreased NRS pain scores at rest and with movement.	The PENG block shows that it is an effective analgesic option in total hip arthroplasty in adults, which shows promise for the applicability in pediatrics as well.
Hu et al. (2024) [37]	Systemic review and meta-analysis of randomized control trials to determine effectiveness of PENG block in relation to opioid use.	Significantly lower opioid consumption in adults 24 h after hip fracture surgery among those who received spinal anesthesia. Significantly lower dynamic pain scores, but no significant difference in static pain scores. PENG block also showed significantly lower risks of unsatisfactory events.	These early trials have evidence that supports PENG blocks can significantly reduce opioid consumption after hip surgery and improve early functional recovery. Still more studies need to be completed in pediatric-specific populations to allow for more solid conclusions.

**Table 2:** Considerations for Choosing PENG vs. Lumbar Epidural vs. Caudal Block in Pediatric Hip Surgery.

Patient/Surgical Factor	Favors PENG Block	Favors Lumbar Epidural	Favors Caudal Epidural
Expected post-op pain severity	Mild–moderate	Moderate–severe	Mild–moderate
Surgical invasiveness	Less invasive procedures	Extensive bilateral or complex surgery	Moderate invasiveness
Need for motor sparing/early mobilization	High priority	Lower priority	Lower priority
Coagulopathy or anticoagulant use	Considered when neuraxial techniques are relatively contraindicated; superficial, extra-neuraxial injection [50]	Contraindicated	Contraindicated

## Data Availability

Data sharing is not applicable to this article as no datasets were generated or analyzed during the current study.

## References

[1] Fan Z., Yan L., Liu H., Li X., Fan K., Liu Q., Li J.J., Wang B. (2023). The Prevalence of Hip Osteoarthritis: A Systematic Review and Meta-Analysis. Arthritis Res. Ther..

[2] Azevedo D.C., Hoff L.S., Kowalski S.C., de Andrade C.A.F., Trevisani V.F.M., de Melo A.K.G. (2024). Risk Factors for Osteoporotic Hip Fracture Among Community-Dwelling Older Adults: A Real-World Evidence Study. Adv. Rheumatol..

[3] Crofts H., McConkey M., Lodhia P. (2023). Pediatric Hip Arthroscopy: A Review of Indications and Treatment Outcomes. Curr. Rev. Musculoskelet. Med..

[4] Cai Y., Lopata L., Roh A., Huang M., Monteleone M.A., Wang S., Sun L.S. (2017). Factors influencing postoperative pain following discharge in pediatric ambulatory surgery patients. J. Clin. Anesth..

[5] Liu S., Genel F., Harris I.A., Patanwala A.E., Adie S., Stevens J., Hassett G., Luckie K., Penm J., Naylor J. (2022). Effectiveness of Pharmacological-Based Interventions, Including Education and Prescribing Strategies, to Reduce Subacute Pain After Total Hip or Knee Arthroplasty: A Systematic Review of Randomized Controlled Trials. Pain Med..

[6] Carlton E.F., Moniz M.H., Scott J.W., Prescott H.C., Becker N.V. (2023). Financial Outcomes After Pediatric Critical Illness Among Commercially Insured Families. Crit. Care.

[7] Daughtrey H.R., Ruiz M.O., Felix N., Saynina O., Sanders L.M., Anand K.J.S. (2024). Incidence of mental health conditions following pediatric hospital admissions: Analysis of a national database. Front. Pediatr..

[8] Odegard M., Kelley-Quon L.I. (2022). Postoperative Opioid Prescribing, Use, and Disposal in Children. Adv. Pediatr..

[9] Kil H.K. (2018). Caudal and epidural blocks in infants and small children: Historical perspective and ultrasound-guided approaches. Korean J. Anesthesiol..

[10] Girón-Arango L., Peng P.W.H., Chin K.J., Brull R., Perlas A. (2018). Pericapsular Nerve Group (PENG) Block for Hip Fracture. Reg. Anesth. Pain Med..

[11] Bunnell A.M., Smith J.V. (2025). Pericapsular Nerve Group (PENG) Block for Pediatric Hip Surgery: A Report of Three Cases. Cureus.

[12] Elhamrawy A., Kerbage J., Veneziano G., Martin D.P., Tobias J.D. (2024). Pericapsular Nerve Group (PENG) Block in Pediatric Patients Undergoing Hip and Pelvic Surgical Procedures: An Educational Focused Review. J. Pain Res..

[13] Turgut M., Protas M., Gardner B., Oskouian R.J., Loukas M., Tubbs R.S. (2017). The accessory obturator nerve: An anatomical study with literature analysis. Anatomy.

[14] Gupta N., Das S., Chatterjee N., Munjal M. (2025). A Retrospective Study of Ultrasound-Guided Pericapsular Nerve Group Block with Dexamethasone: An Excellent Option for Early Mobility Following Total Hip Replacement Surgery. Cureus.

[15] Margenfeld F., Zendehdel A., Poilliot A., Tamborrini G., Beck M., Müller-Gerbl M. (2024). Pericapsular Nerve Group (PENG) Block on Cadavers: A Scoping Review. J. Diagn. Med. Sonogr..

[16] Merella F., Mossetti V. (2020). Ultrasound-guided upper and lower extremity nerve blocks in children. BJA Educ..

[17] Jung Y., Choi S., Lee S., Kim N., Kim E. (2024). Effective protocol for continuous pericapsular nerve group block in femur fracture patients undergoing hip surgery: Two case reports. Kosin Med. J..

[18] Domagalska M., Wieczorowska-Tobis K., Reysner T., Geisler-Wojciechowska A., Grochowicka M., Kowalski G. (2023). Pericapsular Nerves Group (PENG) Block in Children under Five Years of Age for Analgesia in Surgery for Hip Dysplasia: Case Report. J. Pers. Med..

[19] Mostafa T.A.H., Omara A.F., Khalil N.K. (2024). Comparison of ultrasound-guided erector spinae plane block with ultrasound-guided pericapsular nerve group block for paediatric hip surgery: A randomised, double-blinded study. Indian J. Anaesth..

[20] Jadon A., Srivastawa S., Bakshi A., Sahoo R.K., Singh B.K., Sinha N. (2022). Does adding lateral femoral cutaneous nerve block improves the analgesia of pericapsular nerve group block in the fractured hip surgeries?. Braz. J. Anesthesiol..

[21] Balasubramaniam A., Kumar Naggaih S., Tarigonda S., Madhusudhana R. (2023). Ultrasound-Guided Pericapsular Nerve Group Block for Hip Surgery: A Randomized Controlled Trial Study Comparing Ropivacaine and Ropivacaine with Dexamethasone. Cureus.

[22] Kalanjiyam G.P., Kanna R.M., Rajasekaran S. (2023). Pediatric spinal injuries—Current concepts. J. Clin. Orthop. Trauma.

[23] Kao S.C., Lin C.S. (2017). Caudal Epidural Block: An Updated Review of Anatomy and Techniques. BioMed Res. Int..

[24] Jain D., Hussain S.Y., Ayub A. (2022). Comparative evaluation of landmark technique and ultrasound-guided caudal epidural injection in pediatric population: A systematic review and meta-analysis. Paediatr. Anesth..

[25] Xu W., Wei H., Zhang T. (2024). Methods of prolonging the effect of caudal block in children. Front. Pediatr..

[26] Goyal V., Kubre J., Radhakrishnan K. (2016). Dexmedetomidine as an adjuvant to bupivacaine in caudal analgesia in children. Anesth. Essays Res..

[27] Rezayi Soufiani A., Joulani M., Jolani M.S., Parish M. (2024). Accessing the efficacy and peri-operative adverse effects of three different hyperbaric bupivacaine 0.5% dosages for spinal anesthesia induction in lower limb orthopedic surgeries: A randomized clinical trial. BMC Anesthesiol..

[28] Avila Hernandez A.N., Hendrix J.M. (2025). Epidural Anesthesia. StatPearls.

[29] Wani T.M., Dabaliz A., Kadah K., Veneziano G., Tumin D., Tobias J.D. (2020). Comparison of the skin-to-epidural space distance at the thoracic and lumbar levels in children using magnetic resonance imaging. Saudi J. Anesth..

[30] Silva M., Halpern S.H. (2010). Epidural analgesia for labor: Current techniques. Local Reg. Anesth..

[31] Macpherson D., Quondamatteo F., Broom M. (2022). Update on applied epidural anatomy. BJA Educ..

[32] Swezey E., Bordoni B. (2025). Anatomy, Bony Pelvis and Lower Limb: Lateral Femoral Cutaneous Nerve. StatPearls.

[33] Tomlinson J., Ondruschka B., Prietzel T., Zwirner J., Hammer N. (2021). A systematic review and meta-analysis of the hip capsule innervation and its clinical implications. Sci. Rep..

[34] Refai N.A., Black A.C., Tadi P. (2025). Anatomy, Bony Pelvis and Lower Limb: Thigh Femoral Nerve. StatPearls.

[35] Casati A., Santorsola R., Aldegheri G., Ravasi F., Fanelli G., Berti M., Fraschini G., Torri G. (2003). Intraoperative epidural anesthesia and postoperative analgesia with levobupivacaine for major orthopedic surgery: A double-blind, randomized comparison of racemic bupivacaine and ropivacaine. J. Clin. Anesth..

[36] Del Buono R., Padua E., Pascarella G., Costa F., Tognù A., Terranova G., Greco F., Perez M.F., Barbara E. (2021). Pericapsular nerve group block: An overview. Minerva Anestesiol..

[37] Hu X., Chenyang D., Xu B., Lao Y., Sheng H., Zhang S., Huang Y., Chen R.J. (2024). Pericapsular nerve group block reduces opioid use and pain after hip surgery: A systematic review and meta-analysis of randomized controlled trials. PLoS ONE.

[38] Yeoh S.R., Chou Y., Chan S.M., Hou J.D., Lin J.A. (2022). Pericapsular Nerve Group Block and Iliopsoas Plane Block: A Scoping Review of Quadriceps Weakness after Two Proclaimed Motor-Sparing Hip Blocks. Healthcare.

[39] Thomas A., Bhasulamani S.P., James D., Yadav B., Rai E. (2023). A cross-sectional observation study to evaluate the efficacy and complications of epidural analgesia in paediatric population. J. Anaesthesiol. Clin. Pharmacol..

[40] Pearson A.C.S., Dodd S.E., Kraus M.B., Ligda K.M.O., Hertzberg L.B., Patel P.V., Chandrabose R.K. (2019). Pilot Survey of Female Anesthesiologists’ Childbearing and Parental Leave Experiences. Anesth. Analg..

[41] Kasanavesi R.C., Gazula S., Pula R., Thakur N. (2015). Safety of post-operative epidural analgesia in the paediatric population: A retrospective analysis. Indian J. Anaesth..

[42] Bryant J., Joselyn A., Tobias J. (2014). The Complicated Uncomplicated Epidural Placed Under General Anesthesia: A Complete Spinal in the Post-Anesthesia Recovery Unit. J. Med. Cases.

[43] Taenzer A.H., Walker B.J., Bosenberg A.T., Martin L., Suresh S., Polaner D.M., Wolf C., Krane E.J. (2014). Asleep Versus Awake: Does It Matter?. Reg. Anesth. Pain Med..

[44] Wathen J.E., Gao D., Merritt G., Georgopoulos G., Battan F.K. (2007). A randomized controlled trial comparing a fascia iliaca compartment nerve block to a traditional systemic analgesic for femur fractures in a pediatric emergency department. Ann. Emerg. Med..

[45] Remily E.A., Hochstein S.R., Wilkie W.A., Mohamed N.S., Thompson J.V., Kluk M.W., Nace J., Delanois R.E. (2022). The pericapsular nerve group block: A step towards outpatient total hip arthroplasty?. Hip Int..

[46] Wong G.K., Arab A.A., Chew S.C., Naser B., Crawford M.W. (2013). Major complications related to epidural analgesia in children: A 15-year audit of 3,152 epidurals. Can. J. Anesth. J. Can. Anesth..

[47] Orozco S., Muñoz D., Jaramillo S., Herrera A.M. (2019). Pediatric use of Pericapsular Nerve Group (PENG) block for hip surgical procedures. J. Clin. Anesth..

[48] Domagalska M., Ciftci B., Reysner T., Kolasiński J., Wieczorowska-Tobis K., Kowalski G. (2023). Pain Management and Functional Recovery after Pericapsular Nerve Group (PENG) Block for Total Hip Arthroplasty: A Prospective, Randomized, Double-Blinded Clinical Trial. J. Clin. Med..

[49] Sharma H., Mitra S., Singh J., Gupta S., Garg S. (2020). A Randomized Study Comparing the Efficacy of Ultrasound Guided Lumbar Plexus Block and Epidural Anesthesia for Postoperative Analgesia in Patients Undergoing Total Hip Replacement. Asian J. Anesthesiol..

[50] Ecoffey C., Bosenberg A., Lonnqvist P.A., Suresh S., Delbos A., Ivani G. (2022). Practice advisory on the prevention and management of complications of pediatric regional anesthesia. J. Clin. Anesth..

